# Strategies to improve implementation of cascade testing in hereditary cancer syndromes: a systematic review

**DOI:** 10.1038/s41525-024-00412-0

**Published:** 2024-04-03

**Authors:** Jianbang Chiang, Ziyang Chua, Jia Ying Chan, Ashita Ashish Sule, Wan Hsein Loke, Elaine Lum, Marcus Eng Hock Ong, Nicholas Graves, Joanne Ngeow

**Affiliations:** 1 Cancer Genetics Service, Division of Medical Oncology, National Cancer Centre Singapore, Singapore, 169610 Singapore; 2 Oncology Academic Clinical Program, Duke-NUS Medical School, Singapore, Singapore, 169857 Singapore; 3 Lee Kong Chian School of Medicine, Nanyang Technological University, Singapore, 308232 Singapore; 4 Yong Loo Lin School of Medicine, National University of Singapore, Singapore, 117597 Singapore; 5 Health Services & Systems Research, Duke-NUS Medical School, Singapore, 169857 Singapore; 6 Department of Emergency Medicine, Singapore General Hospital, Singapore, 169608 Singapore

**Keywords:** Genetic testing, Health policy

## Abstract

Hereditary cancer syndromes constitute approximately 10% of all cancers. Cascade testing involves testing of at-risk relatives to determine if they carry the familial pathogenic variant. Despite growing efforts targeted at improving cascade testing uptake, current literature continues to reflect poor rates of uptake, typically below 30%. This study aims to systematically review current literature on intervention strategies to improve cascade testing, assess the quality of intervention descriptions and evaluate the implementation outcomes of listed interventions. We searched major databases using keywords and subject heading of “cascade testing”. Interventions proposed in each study were classified according to the Effective Practice and Organization of Care (EPOC) taxonomy. Quality of intervention description was assessed using the TIDieR checklist, and evaluation of implementation outcomes was performed using Proctor’s Implementation Outcomes Framework. Improvements in rates of genetic testing uptake was seen in interventions across the different EPOC taxonomy strategies. The average TIDieR score was 7.3 out of 12. Items least reported include modifications (18.5%), plans to assess fidelity/adherence (7.4%) and actual assessment of fidelity/adherence (7.4%). An average of 2.9 out of 8 aspects of implementation outcomes were examined. The most poorly reported outcomes were cost, fidelity and sustainability, with only 3.7% of studies reporting them. Most interventions have demonstrated success in improving cascade testing uptake. Uptake of cascade testing was highest with delivery arrangement (68%). However, the quality of description of interventions and assessment of implementation outcomes are often suboptimal, hindering their replication and implementation downstream. Therefore, further adoption of standardized guidelines in reporting of interventions and formal assessment of implementation outcomes may help promote translation of these interventions into routine practice.

## Introduction

Approximately 10% of all cancers can be attributed to hereditary cancer syndromes^1^. Yet, they are underdiagnosed currently^2,3^. Hereditary cancer syndromes are a group of conditions which puts an individual at increased risk of developing certain tumors due to an inherited pathogenic variant/likely pathogenic variant (PV/LPV). Most hereditary cancer syndromes are autosomal dominant, where first-degree relatives of the affected patient (proband) have a 1 in 2 (50%) chance to inherit the familial PV/LPV in a cancer susceptibility gene^4^. The care of a patient with a hereditary cancer syndrome extends beyond the affected patient to the family members, as the genetic test results have implications on the rest of the family.

Cascade testing is the process of extending genetic testing to biologic relatives at risk for inheriting a PV/LPV previously identified in an affected patient. Patients are encouraged to discuss cascade testing with at-risk relatives (ARRs)^5^. ARRs can then see a genetic service to undergo germline genetic testing to ascertain if they carry the familial PV/LPV found in the proband. Family members who tested positive for the familial PV/LPV can be made aware of an increased risk of cancer. This allows for implementation of risk management strategies, such as intensive surveillance or risk-reducing procedures, which have the potential to reduce long term morbidity and mortality in this high risk population^6–8^. Over the years, there has been increasing emphasis on cascade testing to identify these ARRs^9,10^. The timely identification of individuals and families with hereditary cancer syndromes can enhance clinician’s suspicion of cancer in view of their inherent elevated risk^11,12^. This impacts surveillance, motivates lifestyle changes, improves personal health choices and affects management plans. Cascade testing for Hereditary Breast and Ovarian Cancer and Lynch syndrome is categorized as a ‘tier 1 genomic application’ by Centres for Disease Control and Prevention (CDC)^13^, which highlights its potential for significant positive impact on public health. International guidelines also encourage testing of ARRs based on its utility for improving health outcomes with early risk management^10,14^. Cascade testing allows the benefits of genetic testing to propagate beyond the affected patients^15,16^, and empower family members to understand their carrier status as well as take charge of their health^17^. Importantly, cascade testing has also been found to be cost-effective in hereditary cancer syndromes, especially with the addition of cascade testing of ARRs^18,19^.

Despite efforts targeted at improving cascade testing uptake, current literature continues to reflect poor rates of uptake, typically below 30%^16,20^. Communication of hereditary cancer syndrome frequently relies on the proband, which may not wish to pass on this personal medical information. Furthermore, poor comprehension of genetics, limited access and concerns about genetic discrimination may further hamper uptake of cascade testing^15,21^. Of note, studies conducted in Asian countries report notably lower rates of cascade testing compared to those in the global community^15,22^. Uptake can be as low as 13%^21^, leaving much room for improvement. In view of the potential benefits of cascade testing, multiple interventions have been attempted to increase referrals for cascade testing in cancer genetic services worldwide^23–25^. While many strategies have shown success in trials, most of these interventions are not integrated into routine practice, failing to achieve their primary endpoint of improving public health. This is commonly referred to as the research-to-practice gap^26^. To close this gap, advances have been made in implementation research, with various tools, checklists and frameworks designed to facilitate replication and ease of implementation^27–29^. An example is the 2011 paper by Proctor and colleagues which described a heuristic taxonomy of eight implementation outcomes to aid in conceptualizing and evaluating success of implementation processes and strategies^27^. A review by Srinivasan et al. discussed interventions, barriers and facilitators to enhance cascade testing, highlighting research gaps including a clear lack of how interventions are implemented, which is important for success of their future application in the public health setting^30^. It has been noted that some of these interventions may work in one healthcare context and not in another^31^. We lack comprehensive information about how these interventions are implemented, and whether these interventions can be applied to unique healthcare settings.

We had three aims for this project. First, to systematically review current literature on intervention strategies to improve cascade testing for hereditary cancer syndromes regarding the quality of intervention descriptions and implementation outcomes of stated interventions. Second, to report the effectiveness of the strategies in measurable clinical outcomes, where available, including number of ARRs referred for genetic counseling and subsequent cascade testing uptake. Lastly, to assess the implementation strategies used to enhance referrals for cascade genetic testing and success of these strategies in terms of implementation outcomes.

## Results

**Fig. 1:**
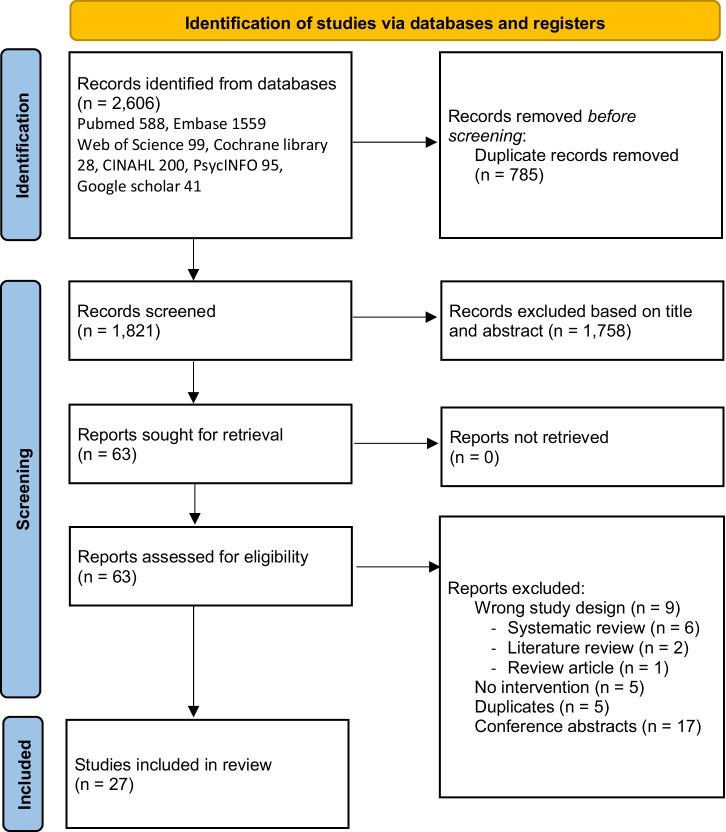
PRISMA 2020 flow chart. Reports excluded (36): wrong study design (9), no interventions (5), duplicates (5),conference abstracts (17).

The database search identified a total of 2606 studies. After title and abstract screening, 63 studies were assessed in full-text screening. Twenty-seven studies were included in the final review (Fig. 1).

### Study characteristics

Study designs in this review include 17 prospective studies, five cross-sectional studies and five retrospective studies. Publication dates ranged from 2013 to 2023 and spanned nine countries. Study characteristics are shown in Table 1. Of the 27 studies included, 17 studies (63.0%) were from the USA, 5 studies (18.5%) did not specify the genes evaluated, and a range of interventions were used. Eight studies (29.6%) evaluated only *BRCA1* and *BRCA2* PV/LPV, three studies (11.1%) looked at Lynch syndrome genes *MLH1, MSH2, MSH6, PMS2* and *EPCAM* PV/LPV, whereas 11 studies (40.7%) evaluated broader gene panels.

**Table 1:** Characteristics of 27 studies included in systematic review

Study author, year	Study title	Country	Study design, sample size of at-risk relatives	Hereditary cancer syndrome	Intervention
Barrow^61^	Improving the uptake of predictive testing and colorectal screening in Lynch syndrome: a regional primary care survey.	UK	Cross sectional study, 591	Lynch syndrome	Enhanced role for GP to facilitate communication within families
Frey^62^	Prospective Feasibility Trial of a Novel Strategy of Facilitated Cascade Genetic Testing Using Telephone Counseling.	USA	Prospective cohort study, 95	Not specified	Facilitated cascade testing via telephone genetic counseling and mailed saliva-based genetic testing
Donenberg^38^	A clinically structured and partnered approach to genetic testing in Trinidadian women with breast cancer and their families.	USA	Prospective cohort study, 125	Breast cancer	A clinically structured and partnered approach
Tone^39^	The Prevent Ovarian Cancer Program (POCP): Identification of women at risk for ovarian cancer using complementary recruitment approaches.	Canada	Prospective cohort study, 564	High grade serous ovarian carcinoma	Outreach and direct recruitment
O’Neil^63^	Information and support needs of young women regarding breast cancer risk and genetic testing: adapting effective interventions for a novel population.	USA	Prospective cohort study, 100	Hereditary breast and ovarian cancer	Peer-coach led telephone counseling
Dilzell^32^	Evaluating the utilization of educational materials in communicating about Lynch syndrome to at-risk relatives.	USA	Retrospective cohort study, 24	Lynch syndrome	Educational materials
Furniss^41^	Novel Models of Genetic Education and Testing for Pancreatic Cancer Interception: Preliminary Results from the GENERATE Study.	USA	Randomized controlled trial, 98	Pancreatic ductal adenocarcinoma	Remote genetic education and testing
Courtney^21^	Impact of free cancer predisposition cascade genetic testing on uptake in Singapore.	Singapore	Prospective cohort study, 826	Not specified	Free cascade testing
Chen^64^	Extended Family Outreach in Hereditary Cancer Using Web-Based Genealogy, Direct-to-Consumer Ancestry Genetics, and Social Media: Mixed Methods Process Evaluation of the ConnectMyVariant Intervention	USA	Prospective cohort study, 57	Not specified	ConnectMy Variant (Web-based genealogy)
Katz^33^	Cascade Genetic Risk Education and Testing in Families With Hereditary Cancer Syndromes: A Pilot Study	USA	Randomized controlled trial, 66	Breast cancer	Online cancer genetic education followed by free or paid ($50) testing
Goodman^65^	Development of a secure website to facilitate information sharing in families at high risk of bowel cancer— The Familyweb Study	UK	Cross-sectional study, 198	Colon cancer	Use of website as a file sharing facility
Li^18^	Impact of subsidies on cancer genetic testing uptake in Singapore	Singapore	Prospective cohort study, 235	Not specified	Subsidy schemes
Schmidlen^24^	Use of a chatbot to increase uptake of cascade genetic testing	USA	Prospective cohort study, 377	Not specified	Cascade chatbot
Garcia^34^	Mechanisms to increase cascade testing in hereditary breast and ovarian cancer: Impact of introducing standardized communication aids into genetic counseling	USA	Prospective cohort study, 40	Hereditary breast and ovarian cancer	Use of communication aids
Aeilts^66^	The impact of a cascade testing video on recipients' knowledge, cognitive message processing, and affective reactions: A formative evaluation.	USA	Cross sectional study, 373	Hereditary breast and ovarian cancer	Use of video-based messaging
Kahn^67^	Barriers to completion of cascade genetic testing: how can we improve the uptake of testing for hereditary breast and ovarian cancer syndrome?	USA	Prospective cohort study, 114	Hereditary breast and ovarian cancer	Follow-up telephone call
Caswell-Jin^23^	Cascade genetic testing of relatives for hereditary cancer risk: Results of an Online Initiative	USA	Prospective cohort study, 2280	Not specified	An online, low-cost family testing program
Patenaude^68^	Young adult daughters of BRCA1/2 positive mothers: What do they know about hereditary cancer and how much do they worry?	USA	Retrospective study, 57	Hereditary breast and ovarian cancer	Professional-family member communication
Yoon^56^	Genetic 3ounselling for patients and families with hereditary breast and ovarian cancer in a developing Asian country: An observational descriptive study	Malaysia	Prospective cohort study, 471	Hereditary breast and ovarian cancer	Cancer genetic counseling service
Haas^69^	Environmental scan of family chart linking for genetic cascade screening in a US integrated health system	USA	Cross-sectional study, N/A	Not specified	Integrating automated family cascade genetic testing into EHR
Frey^70^	What happens in the long term: Uptake of cancer surveillance and prevention strategies among at‐risk relatives with pathogenic variants detected via cascade testing	USA	Prospective cohort study, 95	Not specified	Facilitated cascade testing
Delahunty^71^	TRACEBACK: Testing of Historical Tubo-Ovarian Cancer Patients for Hereditary Risk Genes as a Cancer Prevention Strategy in Family Members.	Australia	Retrospective cohort study, 60	Tubo-ovarian cancer	Retrospective genetic testing in deceased probands
Pande^72^	Development and evaluation of an online, patient-driven, family outreach intervention to facilitate sharing of genetic risk information in families with Lynch syndrome.	USA	Cross sectional study, 56	Lynch Syndrome	FamilyCONNECT online tool
Sermijn^40^	The impact of an interventional counseling procedure in families with a BRCA1/2 gene mutation: efficacy and safety.	Belgium	Prospective cohort study, 172	Hereditary breast and ovarian cancer	Stepwise interventional approach to inform ARRs
Menko^73^	The uptake of predictive DNA testing in 40 families with a pathogenic BRCA1/BRCA2 variant. An evaluation of the proband-mediated procedure.	The Netherlands	Retrospective study, 239	Hereditary breast and ovarian cancer	Guideline containing recommendations regarding proband-mediated procedure
Kassem^74^	Racial Disparities in Family Variant Testing for Cancer Predisposition Genes	USA	Retrospective study, 3872	Not specified	Cascade testing at no-charge
Kauffman^75^	Feasibility of a Traceback Approach for Using Pathology Specimens to Facilitate Genetic Testing in the Genetic Risk Analysis in Ovarian Cancer (GRACE) Study Protocol	USA	Prospective cohort study, N/A	Ovarian cancer	Traceback approach for using pathology specimens

*N/A* not applicable.

### Taxonomy of health systems interventions

Intervention components were mapped to an adapted EPOC taxonomy. Some studies described multicomponent interventions without differentiating between individual components’ efficacy. We ascertained the primary intervention as the intervention of interest. Out of 27 studies, proposed interventions in 20 studies (74.1%) were classified into delivery arrangements, of which 11 were categorized under “Information and communication technology” and nine under “Coordination of care and management of care processes”. Four studies (14.8%) evaluated implementation strategies, of which all four were categorized into interventions targeted at healthcare workers. Three studies (11.1%) attempted to address financial arrangements, of which all fall under the category of collection of funds. A summary is presented in Table 2.

**Table 2:** Classification of interventions reported in included studies based on EPOC taxonomy strategies and categories and reported rate of uptake of genetic testing for the post-intervention and control group

EPOC taxonomy strategy	Study	EPOC taxonomy category	Intervention	Rate of uptake of genetic testing post-intervention/%	Rate of uptake of genetic testing for control group/%
Delivery arrangements	Barrow^61^	Coordination of care and management of care processes	Enhanced role for GP to facilitate communication within families	–	–
	Donenberg^38^		Family counseling session by genetic counselor with local management team within 14 days of initial visit, with free single-site genetic testing.	99.0	–
	Tone^39^		Two recruitment methods. 1. Outreach approach - clinician education and media campaigns to direct potential participants to a study website 2. Direct recruitment – letter was mailed to the deceased’s family physician to notify ARR	93.3	–
	Dilzell^32^		Utilization of educational materials - Genetic counseling note, family letter, personal note from proband, information/report from laboratory, online resource, support group information, referral to genetics clinic	51.0	19.0
	Kahn^67^		Follow-up telephone call after 6 months for ARR who reported interest in genetic testing but did not return saliva kit	35.7	–
	Yoon^56^		Cancer genetic counseling session	11	–
	Delahunty^71^		Retrospective genetic testing in deceased probands, with contact of ARR	–	–
	Sermijn^40^		stepwise interventional approach to inform ARR. Phase I - proband informed ARR. Phase II (after 6 months) - letter sent to ARR Phase III - phone call to obtain a final decision.	97.8	–
	Kauffman^75^		Traceback approach by using pathology specimens to identify patients with ovarian cancer and offering genetic testing to them and ARR	–	–
	Frey^62^	Information and communication technology (ICT)	Direct telephone contact of ARR by the genetics team, with telephone genetic counseling. Mailed saliva kit for genetic testing was provided free of charge. Telephone disclosure of genetic test results, with release of results to primary care physician	70.0	–
	O’Neil^63^		Three sessions of peer-coach lead telephone counseling	–	–
	Furniss^41^		Remote genetic education and testing	92.0	–
	Katz^33^		Online cancer genetic education followed by free or paid genetic testing	83.3	94.4
	Goodman^65^		The use of a website as a web-based file sharing facility (Family Web website)	–	–
	Schmidlen^24^		family sharing tool and chatbot	–	–
	Aeilts^66^		2 minute animated video for proband to share with ARR	–	–
	Caswell-Jin^23^		An online, low-cost family testing program	47.5	–
	Haas^69^		Integrating automated family cascade genetic testing into electronic health records	–	–
	Frey^70^		Direct telephone contact of ARRs made by genetics team	70	–
	Pande^72^		FamilyCONNECT online tool	–	–
Financial arrangements	Courtney^21^	Collection of funds	free cascade testing	21.6	6.1
	Li^18^		Subsidy schemes -blanket and varied schemes	53.3	47.5
	Kassem^74^		providing predictive testing for ARR at no-charge	–	–
Implementation strategies	Chen^64^	Interventions targeted at healthcare workers	ConnectMyVariant intervention to provide educational information on how to spread awareness among families	–	–
	Garcia^34^		Use of educational resources as a supplement to genetic counseling.	4.5	0
	Patenaude^68^		Healthcare professional-family member communication	–	–
	Menko^73^		Dutch guideline containing recommendations for facilitating proband-mediated disclosure	43	–

*GP* general practitioner, *ARR* at-risk relatives.

Among the 20 studies under delivery arrangements, two studies reported uptake rates of genetic testing pre- and post-intervention. Dilzell et al. evaluated the use of educational materials which led to a higher uptake post-intervention (51%) as compared to control, where no materials were used (19%)^32^. On the other hand, Katz et al. investigated on the effect of free genetic testing which reflected a lower rate of uptake post-intervention (83.3%) as compared to control which received low-cost testing (94.4%)^33^. Nine studies reported rate of uptake of genetic testing post-intervention only, of which six reported uptake rates of 70% and above, reflecting relatively high rates of genetic testing.

Among the four studies under implementation strategies, one study reported rate of genetic testing uptake post-intervention and control. Garcia et al. evaluated the use of communication aids which reported a higher uptake post-intervention (4.5%) as opposed to control (0%)^34^. One other study reported rate of genetic testing uptake post-intervention only. This study by Menko et al. investigated the outcomes from implementation of guidelines by the Dutch Society for Clinical Genetics on proband-mediated dissemination of genetic information via proband education, family letters and follow-up phone call. The study reported a 43% uptake rate for genetic testing^35^.

Among the three studies targeting financial arrangements, two studies reported rate of genetic testing uptake post-intervention and control. Courtney et al. studied the impact of free cascade testing while Li et al. looked at the efficacy of subsidy schemes^18,21^. Both studies reported a higher uptake post-intervention as opposed to control, with the former reporting rates of 21.6% vs 6.1%, and the latter 53.3% vs 47.5%.

### Quality of description of intervention strategies

The mean TIDieR score for the 27 included studies was 7.3 out of 12. Six items were reported in more than 80% of the studies; these include (1) brief name of intervention (100%), (2) intervention rationale (96.3%), (3) intervention providers (88.9%), (4) description of procedures (85.2%), (5) description of materials (85.2%), (6) frequency/ timing, dose, duration (85.2%). Fewer than 20% of studies reported these items: (1) modifications (18.5%), (2) plans to assess adherence/fidelity (7.4%), (3) actual assessment of fidelity/adherence (7.4%). None of the studies provided detailed descriptions of all 12 items on the TIDieR checklist. A summary is presented in Table 3.

**Table 3:** Quality of description of intervention strategies based on the TIDieR checklist

Study	TIDieR items												TIDieR score^a^
	1.Brief name of intervention	2.Intervention rationale	3.Description of materials	4.Description of procedures	5.Intervention provider	6.Mode of delivery	7.Location	8.Frequency/ timing, dose, duration, item	9.Tailoring	10.Modifications	11.Plans to assess adherence/ fidelity	12.Actual assessment of fidelity/adherence	
Barrow^61^	✓	✓			✓								3
Frey^62^	✓	✓	✓	✓	✓	✓	✓	✓	✓				9
Donenberg^38^	✓	✓	✓	✓	✓	✓		✓					7
Tone^39^	✓	✓	✓	✓	✓	✓	✓	✓					8
O’Neil^63^	✓	✓	✓	✓	✓	✓		✓		✓			8
Dilzell^32^	✓	✓	✓		✓			✓					5
Furniss^41^	✓	✓	✓	✓	✓	✓	✓	✓	✓				9
Courtney^21^	✓	✓	✓	✓	✓		✓	✓					7
Chen^64^	✓	✓	✓	✓	✓	✓	✓	✓		✓	✓	✓	11
Katz^33^	✓	✓	✓	✓	✓	✓	✓	✓					8
Goodman^65^	✓	✓	✓	✓	✓	✓	✓	✓	✓	✓			10
Li^18^	✓	✓		✓	✓			✓	✓				6
Schmidlen^24^	✓	✓	✓	✓	✓	✓		✓	✓		✓	✓	10
Garcia^34^	✓	✓	✓	✓				✓					5
Aeilts^66^	✓	✓	✓	✓	✓	✓		✓					7
Kahn^67^	✓	✓	✓	✓	✓	✓		✓					7
Caswell-Jin^23^	✓		✓	✓	✓	✓		✓					6
Patenaude^68^	✓	✓								✓			3
Yoon^56^	✓	✓	✓	✓	✓	✓		✓	✓				8
Haas^69^	✓	✓	✓		✓	✓							6
Frey^70^	✓	✓	✓	✓	✓	✓	✓	✓	✓				9
Delahunty^71^	✓	✓	✓	✓	✓	✓	✓	✓	✓				9
Pande^72^	✓	✓	✓	✓	✓	✓	✓			✓			8
Sermijn^40^	✓	✓	✓	✓	✓	✓	✓	✓					8
Menko^73^	✓	✓	✓	✓		✓		✓					6
Kassem^74^	✓	✓		✓	✓	✓		✓					6
Kauffman^75^	✓	✓	✓	✓	✓	✓	✓	✓					8
No. of studies with adequate description	27	26	23	23	24	21	12	23	9	5	2	2	
Percentage of studies with adequate description/ %	100	96.3	85.2	85.2	88.9	77.8	44.4	85.2	33.3	18.5	7.4	7.4	

^a^We allocated one point for each item of the TIDieR checklist to indicate completeness of the descriptions of strategies.

### Implementation outcomes

Of the eight aspects of implementation, an average of 2.9 aspects were evaluated. No single study evaluated all eight implementation outcomes - acceptability, adoptions, appropriateness, feasibility, fidelity, implementation cost, penetration and sustainability. Majority of the studies reported on feasibility (21/27, 77.8%), appropriateness (18/27, 66.7%) and penetration (16/27, 59.1%) of the interventions. Slightly below half studied acceptability (12/27, 44.2%). The least commonly reported outcomes were cost, fidelity and sustainability, with only 3.7% (1/27) of studies reporting them.

The penetration of the intervention, defined as the proportion of participants who took part in the intervention with respect to the total eligible population, varied widely from 10% to 100%, with an average penetration of 52.4% amongst the studies. A summary is presented in Table 4.

**Table 4:** Implementation outcomes based on Proctor’s Implementation Outcomes Framework

Study	Acceptability	Appropriateness	Adoption	Cost	Feasibility	Fidelity	Penetration	Sustainability
Barrow^61^		✓	✓					
Frey^62^	✓	✓			✓		✓	
Donenberg^38^	✓	✓			✓		✓	
Tone^39^			✓		✓		✓	
O’Neil^63^	✓	✓			✓		✓	
Dilzell^32^		✓						
Furniss^41^		✓			✓		✓	
Courtney^21^		✓	✓		✓		✓	
Chen^64^		✓			✓		✓	
Katz^33^		✓			✓		✓	
Goodman^65^	✓					✓		
Li^18^		✓	✓	✓	✓			
Schmidlen^24^		✓			✓		✓	
Garcia^34^	✓				✓			
Aeilts^66^	✓	✓			✓		✓	
Kahn^67^					✓		✓	
Caswell-Jin^23^					✓			
Patenaude2013^68^								
Yoon^56^	✓				✓		✓	
Haas^69^	✓	✓	✓					
Frey^70^	✓				✓		✓	
Delahunty^71^	✓	✓	✓		✓		✓	
Pande^72^	✓	✓	✓					
Sermijn^40^	✓	✓	✓		✓		✓	
Menko^73^		✓			✓		✓	✓
Kassem^74^					✓			
Kauffman^75^		✓			✓			

(✓) indicates outcome was described.

## Discussion

Our study systematically evaluated interventions to enhance cascade testing, ascertained rates of improved uptake and assessed them based on implementation outcomes. This systematic review highlights the success of several intervention efforts to increase cascade testing for hereditary cancer syndrome in family members, but also a clear lack of an implementation science approach in propagation of these successful interventions.

Genetic testing has become mainstream, with increasing number of patients being referred for genetic testing for treatment indications^36,37^. In the same vein, with more patients identified with hereditary cancer syndromes, there ought to be a corresponding increase in identification of ARRs. Overall, most interventions have demonstrated success in improving cascade testing uptake. This success is seen across the different EPOC taxonomy strategies. Amongst the studies that provided uptake information, the mean uptake is 41% in the intervention group compared with 33% in the control group. Uptake of cascade testing was highest with delivery arrangement (68%), compared to financial arrangement (37%) and implementation strategies (24%). There is a large difference in uptake as the success of an intervention does not just depend on the intervention, but also its implementation. Studies that have shown prominent success of more than 90% uptake post intervention often incorporate a multi-tiered approach with appropriate facilitation to ensure optimal implementation. Donenberg et al. integrated a local management team with the genetics team and ensured that the family counseling session occurred within two weeks, with free predictive testing^38^. Tone et al. used a two pronged approach with both outreach to the general public and direct recruitment of patients via their physician to achieve testing rates of 93.3%^39^. Sermijin utilized a three-step approach to inform ARRs via the proband, sending letters and a telephone call to follow up by the genetics team^40^. Furniss et al. improved genetic testing through convenience, allowing remote genetic education with a telemedicine platform and saliva-based genetic testing coordinated by the genetics team^41^. On the other hand, interventions with poor success rates were often one-dimensional, with use of a single genetic counseling session or providing supplementary educational materials with no further input from the genetics team. The distribution across the taxonomy strategies was largely in favor of delivery arrangements (20/27, 74%), while implementation strategies and financial arrangements formed 15% (4/27) and 11% (3/27) of the studies respectively. This suggests that most studies focus on individual tools such as educational materials, websites, targeted at individual patients or healthcare providers to improve cascade testing uptake. Generally, information and communication technology was most frequently used since technology-enabled care has been shown to be noninferior to in-person counseling, and is in fact more accessible and cost-effective^42^. Technology-enabled care requires an appropriate infrastructure^12,43^, which may be feasible in developed countries with a well-established communication network. There is minimal focus on how interventions can be integrated within existing healthcare pathways. Healthcare systems may need to adapt the intervention to suitably assimilate into the local setting, with follow up to ensure appropriate improved outcomes^44^. Further exploration of factors such as implementation and cost may allow more seamless integration of interventions within healthcare organizations.

Increasing specialization in the medical field has resulted in fragmented care for the patient^45^, and in this case, his/ her family. Based on our study, coordination of care and management of care processes is the best form of intervention to improve cascade testing rates for families with hereditary cancer syndromes, with three studies showing post intervention uptake rates above 90%. It is important to recognize the importance of healthcare infrastructure on coordinated intervention efforts^46^, and the success of interventions may not be portable across health systems without adaptation. Several included studies incorporated direct contact of relatives by healthcare staff, but in practice this is limited by privacy laws prohibiting disclosure of genetic information to a third party without proband consent^47^. Families desire support from healthcare professionals in conveying hereditary genetic risk information, and this direct approach is acceptable to relatives^25^. This was echoed in a recent meta-analysis which confirmed that direct relative contact increases rates of cascade genetic counseling and testing^20^, and argued for current privacy laws and infrastructure to be revisited. Future studies may consider breaking down these groups of healthcare professionals to better understand the impact on uptake of cascade testing when facilitated by different types of healthcare professionals. We observed that most studies evaluated at-risk relatives as a congregate, without differentiating into first- or second-degree relatives. Such information could potentially be useful for informing future implementation studies. While no included studies evaluated government interventions, the effects of legalizing disclosure to ARRs even without probands’ consent as in New South Wales, Australia should be monitored^48^, bearing in mind the ongoing debate between healthcare professionals’ duty of care to ARRs and duty of confidentiality to the proband.

Our review also illustrates that implementation outcomes are often selectively evaluated. Feasibility, appropriateness and penetration are outcomes most frequently examined, while cost, fidelity and sustainability are often overlooked. Cost is often a factor that is cited by studies as a barrier to cascade testing^18,49^. Three out of 27 studies evaluated cost and showed that offering free cascade testing can remove a significant barrier, but this requires either further investment in a budget-constrained healthcare system or third-party payers. Additionally, the cost of implementation in the real world is not reported in the majority of included studies. A previous review by Allen et al. reported feasibility and appropriateness as the most frequently measured outcomes^50^. Another review by Proctor also reported cost and sustainability to be the least studied. Hence, the findings from our review largely supports prevailing literature^51^. Notably, sustainability was evaluated in only one study. This was likely due to the high cost of maintaining data collection beyond the study period. However, sustainability is a key aspect of implementation^52^, as it ascertains if the intervention was integrated into practice, the primary end goal for most interventions. The omission of key aspects underscores the need for increased utilization of implementation science frameworks in the evaluation of outcomes to increase cascade testing uptake. Formal assessment of implementation outcomes can aid stakeholders in making fair comparisons among interventions and ultimately adopt the one most relevant to their population. Given such varying extents of implementation outcome reporting, further work is needed to educate healthcare professionals on applying methods for implementing and reporting novel interventions. Implementation outcomes should be formally assessed to ensure these interventions have meaningful, long-lasting impact on the care of patients and ARRs at increased risk of cancer.

Our review highlights the lack of standardization in the reporting of interventions, as shown by inadequate intervention description. The mean TIDieR score for the 27 included studies was 7.3 out of 12, implying only slightly above half of the intervention characteristics were described adequately. This is concerning as it has been well-documented that poor descriptions of interventions may pose a serious challenge to the scientific community in the replication of interventions^53,54^. In this review, one of the most commonly omitted item was modifications made. The reporting of modifications is undeniably important given that certain alterations may have been made during the study to overcome an unexpected difficulty or to achieve better recruitment. Consequently, it appears that there was little tailoring to individuals or modification in a vast majority of the included trials. Tailoring during the study, which may be necessary in cascade testing where relative’s knowledge of hereditary cancer syndrome may not be uniform and will likely require bespoke communication strategies to best fit the participant^55^. Failure to report these details may affect the replication and implementation downstream, preventing the implemented interventions from achieving their desired outcomes. Hence, further work is needed to encourage more widespread adoption of standardized guidelines in reporting of interventions.

Our review has several limitations. The EPOC taxonomy uses categories with some overlap so some interventions could fit into multiple categories, a limitation recognized by its authors. In these circumstances, we chose the classification that best fit the intent of the intervention in the context of our research question, i.e. the means by which the intervention aimed to increase uptake of cascade testing or genetic counseling. A majority of the studies included were targeted at participants in the USA, hence the findings may not be generalizable to Asian countries, where the rates of genetic testing and disclosure to family members have been reported to be significantly lower compared to European families^18,56,57^. Application of insights should be guided by knowledge of cultural and societal factors. Future reviews can consider evaluating the success of intervention strategies trialed and tested solely among Asian populations.

In conclusion, while there are many potentially efficacious strategies devised, further improvement in the reporting quality of studies in this field may be crucial to close the research-to-practice gap. Applying implementation science is therefore essential to ensure effective translation of intervention strategies that increase cascade testing from the experimental to public health setting. This review revealed that while interventions demonstrate effectiveness in experimental settings, we lack robust evaluation of implementation of interventions to optimize uptake of cascade testing. Moving forward, standardized reporting guidelines such as Standards for Reporting Implementation Studies (StaRI) should be used and implementation outcomes formally assessed to ensure interventions have meaningful, lasting impact on patients and relatives, within and beyond cancer genetics.

## Methods

We followed the Preferred Reporting Items for Systematic Reviews and Meta-Analyses (PRISMA) guidelines for good reporting^58^.

### Search strategy

We searched PubMed, Embase (Elsevier), Web of Science, Cochrane Library, CINAHL, PsycINFO, and Google scholar using keywords and subject headings including “cascade testing” or synonymous terms. Search strategies were refined in consultation with a university librarian. Complete search strategy for PubMed and other databases are available (Supplementary Table 1). Peer-reviewed articles published in English between 1 January 2010 and 30 June 2022 were selected. This timeframe reflects current interventions as panel genetic testing has become more common in the past decade^59^, with increasing public acceptance^60^ and new genetic privacy laws^47^. Backward and forward reference searching was conducted for included studies. References were uploaded to Covidence (www.covidence.org), a systematic review management software. All procedures followed were in accordance to the Declaration of Helsinki.

### Eligibility criteria

Study selection is summarized in Fig. 1. We included studies on interventions that target patients with a hereditary cancer syndrome, harboring a PV/LPV in a cancer susceptibility gene. These studies included interventions aimed at improving cascade testing uptake or genetic counseling referral rates. Interventions with multiple components were included. Our review included original papers with quantitative, qualitative and mixed methods study designs and excluded non-English, and non-peer reviewed publications (Supplementary Table 2).

### Study extraction and synthesis

Two reviewers (JC, ZC) separately screened each title and abstract for eligibility after duplicates were removed. These reviewers were blinded to the screening decisions made by the other and could only view their own screening decisions. Disagreements were resolved through discussion between reviewers with adjudication by a third senior reviewer (JN) when a consensus could not be reached. The same process was performed for the full-text review. A data extraction form was developed by the author (JC), then reviewed by all the members of the study team. The standardized form was used for data extraction by three reviewers (CJY, AAS, LWH). Reviewers piloted data extraction using two papers to ensure consistency in approach prior to full data extraction.

### Study appraisal and assessment

Interventions proposed in each study were grouped based on the Effective Practice and Organization of Care (EPOC) taxonomy and the rate of uptake of genetic testing were recorded to determine the efficacy of interventions. The 12-item Template for Intervention Description and Replication (TIDieR) checklist was used to evaluate quality and completeness of intervention description in the included studies. The TIDieR score was calculated for each intervention by summing the number of items reported. Proctor’s Implementation Outcomes Framework was used to assess the implementation outcomes^27^. The eight outcomes assessed include acceptability, appropriateness, adoption, feasibility, fidelity, cost, penetration and sustainability.

### Supplementary information

Supplementary Information

## Data Availability

Data is available upon reasonable request.
