# Supplementary material for: Strategies to improve implementation of cascade testing in hereditary cancer syndromes: a systematic review
Source: NPJ Genom Med. 2024 Apr 3;9:26. doi: 10.1038/s41525-024-00412-0 (PMC10991315; doi:10.1038/s41525-024-00412-0)
Supplement: Supplementary file 1 — Supplementary Information [file 41525_2024_412_MOESM1_ESM.pdf]

Supplementary Information

Supplementary Table 1. Search strategy for databases

Database (date searched)	Query	Search strategy	No. of results
PubMed (9 Nov 22)	1	"neoplastic syndromes, hereditary"[MeSH Terms]	56586
	2	(cancer*[Title/Abstract] OR neoplasm*[Title/Abstract] OR neoplastic[Title/Abstract]) AND (hereditary[Title/Abstract] OR predispos*[Title/Abstract] OR inherit*[Title/Abstract] OR familial[Title/Abstract])	52371
	3	#1 OR #2	99813
	4	"genetic testing"[MeSH Terms:noexp]	43802
	5	"family test*"[Title/Abstract] OR "family screen*"[Title/Abstract] OR "family variant test*"[Title/Abstract] OR "family genetic test*"[Title/Abstract] OR “cascade test*”[Title/Abstract]” OR "cascade screen*"[Title/Abstract] OR “cascade genetic test*”[Title/Abstract]” OR "cascade genetic screen*"[Title/Abstract] OR "predictive screen*"[Title/Abstract] OR "predictive genetic test*"[Title/Abstract]	2976
	6	#4 OR #5	45841
	7	"referral and consultation"[MeSH Terms]	84140
	8	referral*[Title/Abstract] OR "second opinion*"[Title/Abstract] OR "consult*"[Title/Abstract] OR "appointment*"[Title/Abstract] OR "schedule*"[Title/Abstract] OR "uptake"[Title/Abstract] OR "decision making"[Title/Abstract] OR "decision-making"[Title/Abstract] OR "decisionmaking"[Title/Abstract]	1030351
	9	#7 OR #8	1066918
	10	#3 AND #6 AND #9	802
	11	#3 AND #6 AND #9 Filters: from 2010 – 2022	588
Embase (24 Aug 22)	1	exp hereditary tumor syndrome/	57165
	2	(cancer* or neoplasm* or neoplastic).ab,ti.	3137278
	3	(hereditary or predispos* or inherit* or familial).ab,ti.	568700
	4	2 and 3	73818
	5	1 or 4	119588
	6	*genetic screening/	20103
	7	("cascade test*" or "family test*" or "family variant test*" or "cascade screen*" or "family screen*" or "predictive screen*" or "predictive genetic test*" or "cascade genetic test*" or "cascade genetic screen*").ab,ti.	4753
	8	6 or 7	24178
	9	exp patient referral/	141365
	10	(referral* or "second opinion*" or "consult*" or "health service gatekeep*" or "appointment*" or "Schedule*" or "uptake" or "decision making" or "decision-making").ab,ti.	1422181
	11	9 or 10	1479438

	12	5 and 8 and 11	627
	13	5 and 8 and 11<br>limit 12 to yr="2010 - 2022"	551
Web of Science (26 Aug 22)	1	TS=("neoplastic syndromes, hereditary")	17
	2	TS=((cancer* OR neoplasm* OR neoplastic) AND (hereditary OR predispos* OR inherit* OR familial))	65440
	3	#1 OR #2	65440
	4	TS="genetic testing"	28403
	5	TS=("cascade test*" OR "family test*" OR "family variant test*" OR "cascade screen*" OR "family screen*" OR "predictive screen*" OR "predictive genetic test*" OR "cascade genetic test*" OR "cascade genetic screen*")	3316
	6	#4 OR #5	30572
	7	TS=("referral and consultation")	952
	8	TS=(referral* OR "second opinion*" OR "consult*" OR "health service gatekeep*" OR "appointment*" OR "Schedule*" OR "uptake" OR "decision making" OR "decision-making")	1634013
	9	#7 OR #8	1634013
	10	#3 AND #6 AND #9	1018
		#3 AND #6 AND #9 and 2010 or 2011 or 2012 or 2022 or 2021 or 2020 or 2019 or 2018 or 2017 or 2016 or 2015 or 2014 or 2013 (Publication Years)	753
Cochrane Library (29 Aug 22)	1	[mh "neoplastic syndromes, hereditary"]	535
	2	(cancer*:ti,ab OR neoplasm*:ti,ab OR neoplastic:ti,ab) AND (hereditary:ti,ab OR predispos*:ti,ab OR inherit*:ti,ab OR familial:ti,ab)	1223
	3	#1 OR #2	1667
	4	[mh ^"genetic testing"]	422
	5	(cascade NEXT test*):ti,ab OR (family NEXT test*):ti,ab OR ("family variant" NEXT test*):ti,ab OR (cascade NEXT screen*):ti,ab OR (family NEXT screen*):ti,ab OR (predictive NEXT screen*):ti,ab OR ("predictive genetic" NEXT test*):ti,ab OR ("cascade genetic" NEXT test*):ti,ab OR ("cascade genetic" NEXT screen*):ti,ab	71
	6	#4 OR #5	486
	7	[mh "referral and consultation"]	2543
	8	referral*:ti,ab OR (second NEXT opinion*):ti,ab OR consult*:ti,ab OR ("health service" NEXT gatekeep*):ti,ab OR appointment*:ti,ab OR Schedule*:ti,ab OR uptake:ti,ab OR "decision making":ti,ab OR decision-making:ti,ab	121518
	9	#7 OR #8	122266
	10	#3 AND #6 AND #9	40
	11	#3 AND #6 AND #9<br>with Cochrane Library publication date from Jan 2010 to Jun 2022	29
CINAHL (29 Aug 22)	1	MH("neoplastic syndromes, hereditary"+)	1138
	2	((TI cancer* OR AB cancer*) OR (TI neoplasm* OR AB neoplasm*) OR (TI neoplastic OR AB neoplastic)) AND ((TI	5582

		hereditary OR AB hereditary) OR (TI predispos* OR AB predispos*) OR (TI inherit* OR AB inherit*) OR (TI familial OR AB familial))	
	3	S1 OR S2	6771
	4	MH("genetic testing")	1105
	5	(TI "cascade test*" OR AB "cascade test*") OR (TI "family test*" OR AB "family test*") OR (TI "family variant test*" OR AB "family variant test*") OR (TI "cascade screen*" OR AB "cascade screen*") OR (TI "family screen*" OR AB "family screen*") OR (TI "predictive screen*" OR AB "predictive screen*") OR (TI "predictive genetic test*" OR AB "predictive genetic test*") OR (TI "cascade genetic test*" OR AB "cascade genetic test*") OR (TI "cascade genetic screen*" OR AB "cascade genetic screen*")	787
	6	S4 OR S5	1382
	7	(MH "referral and consultation"+)	124
	8	(TI referral* OR AB referral*) OR (TI "second opinion*" OR AB "second opinion*") OR (TI consult* OR AB consult*) OR (TI "health service gatekeep*" OR AB "health service gatekeep*") OR (TI appointment* OR AB appointment*) OR (TI Schedule* OR AB Schedule*) OR (TI uptake OR AB uptake) OR (TI "decision making" OR AB "decision making") OR (TI decision-making OR AB decision-making)	286122
	9	S7 OR S8	286181
	10	S3 AND S6 AND S9	17
	11	S3 AND S6 AND S9<br>Limiters - Published Date: 20100101-20220631	17
PsycINFO (29 Aug 22)	1	MH("neoplastic syndromes, hereditary"+)	81
	2	((TI cancer* OR AB cancer*) OR (TI neoplasm* OR AB neoplasm*) OR (TI neoplastic OR AB neoplastic)) AND ((TI hereditary OR AB hereditary) OR (TI predispos* OR AB predispos*) OR (TI inherit* OR AB inherit*) OR (TI familial OR AB familial))	1737
	3	S1 OR S2	3249
	4	MH("genetic testing")	1
	5	(TI "cascade test*" OR AB "cascade test*") OR (TI "family test*" OR AB "family test*") OR (TI "family variant test*" OR AB "family variant test*") OR (TI "cascade screen*" OR AB "cascade screen*") OR (TI "family screen*" OR AB "family screen*") OR (TI "predictive screen*" OR AB "predictive screen*") OR (TI "predictive genetic test*" OR AB "predictive genetic test*") OR (TI "cascade genetic test*" OR AB "cascade genetic test*") OR (TI "cascade genetic screen*" OR AB "cascade genetic screen*")	352
	6	S4 OR S5	353
	7	(MH "referral and consultation"+)	32
	8	(TI referral* OR AB referral*) OR (TI "second opinion*" OR AB "second opinion*") OR (TI consult* OR AB consult*) OR (TI "health service gatekeep*" OR AB "health service gatekeep*") OR (TI appointment* OR AB appointment*) OR (TI Schedule*	246901

		OR AB Schedule*) OR (TI uptake OR AB uptake) OR (TI "decision making" OR AB "decision making") OR (TI decision-making OR AB decision-making)	
	9	S7 OR S8	246913
	10	S3 AND S6 AND S9	12
	11	S3 AND S6 AND S9<br>Limiters - Publication Year: 2010-2022	7
Google Scholar (26 Aug 22)	1	cancer|neoplasm hereditary|familial "cascade test"|"family variant test"|"cascade screen"|"family screen"|"predictive screen"|"predictive genetic test"|"cascade genetic test"|"cascade genetic screen" referral|consult|uptake|"decision making"	861
		Filter: 2010 - 2022	489

Supplementary Table 2. Study inclusion and exclusion criteria

Included studies	
Qualitative studies	Randomized controlled trials, controlled before and after studies, interrupted time series analyses, prospective or retrospective cohort studies, case-control studies, and cross-sectional studies that explain any kind of intervention that pertains to cascade genetic testing and testing uptake
Qualitative studies	Individual interviews, focus group discussions, ethnographic interviews and participant observation, which investigated the implementation of interventions related to genetic screening and testing or which documented people's experiences with those interventions
Mixed methods studies	Interaction of process and implementation factors with engagement in cascade genetic testing uptake
Excluded studies	
Reviews	Literature reviews, scoping reviews and systematic reviews
Others	Non-peer reviewed publications, conference abstracts, editorials, and opinion pieces.
Other languages	Non-English publications
